# Older adults use fewer muscles to overcome perturbations during a seated locomotor task

**DOI:** 10.1152/jn.00263.2023

**Published:** 2024-05-08

**Authors:** Seyed Yahya Shirazi, Helen J. Huang

**Affiliations:** ^1^Swartz Center for Computational Neuroscience, Institute for Neural Computation, University of California San Diego, La Jolla, California, United States; ^2^Department of Mechanical and Aerospace Engineering, University of Central Florida, Orlando, Florida, United States; ^3^Disability, Aging and Technology (DAT) Cluster, University of Central Florida, Orlando, Florida, United States

**Keywords:** aging, motor adaptation, use-dependent learning

## Abstract

Locomotor perturbations provide insights into humans’ response to motor errors. We investigated the differences in motor adaptation and muscle cocontraction between young and older adults during perturbed-arm and -leg recumbent stepping. We hypothesized that besides prolonged adaptation due to use-dependent learning, older adults would exhibit greater muscle cocontraction than young adults in response to the perturbations. Perturbations were brief increases in resistance applied during each stride at the extension onset or midextension of the left or right leg. Seventeen young adults and eleven older adults completed four 10-min perturbed stepping tasks. Subjects were instructed to follow a visual pacing cue, step smoothly, and use all their limbs to drive the stepper. Results showed that young and older adults did not decrease their errors with more perturbation experience, and errors did not wash out after perturbations were removed. Interestingly, older adults consistently had smaller motor errors than young adults in response to the perturbations. Older adults used fewer muscles to drive the stepper and had greater cocontraction than young adults. The results suggest that, despite similar motor error responses, young and older adults use distinctive muscle recruitment patterns to perform the motor task. Age-related motor strategies help track motor changes across the human life span and are a baseline for rehabilitation and performance assessment.

**NEW & NOTEWORTHY** Older adults often demonstrate greater cocontraction and motor errors than young adults in response to motor perturbations. We demonstrated that older adults reduced their motor errors more than young adults with brief perturbations during recumbent stepping while maintaining greater muscle cocontraction. In doing so, older adults largely used one muscle pair to drive the stepper, tibialis anterior and soleus, whereas young adults used all muscles. These two muscles are crucial for maintaining upright balance.

## Graphical abstract

**Figure:**
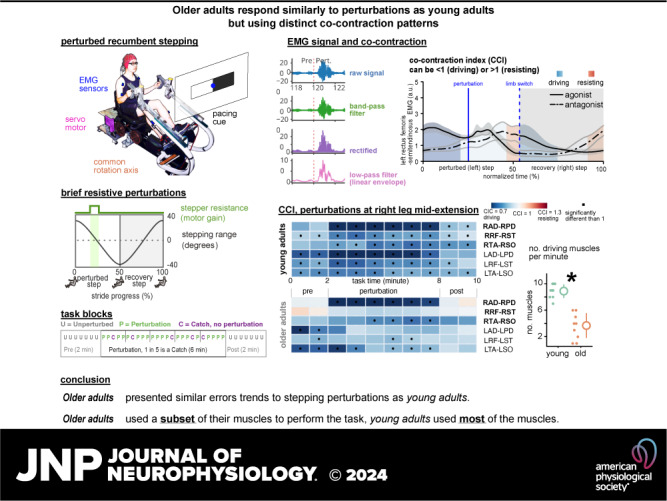

## INTRODUCTION

Muscle cocontraction, i.e., the concurrent activity of the agonist and antagonist muscles, is a common strategy when responding to motor perturbations and during increased uncertainty. This cocontraction usually decreases with the progression of adaptation and reduction of motor errors in response to the perturbations (1, 2). In upper limb reaching tasks, young adults use cocontraction strategically to adapt rapidly to perturbations and improve accuracy (3, 4). In lower limb balance and locomotor tasks, young adults initially increase muscular activity in response to postural balance challenges and during split-belt walking (5, 6). This muscular activity gradually decreases after adaptation to the perturbations and with decreasing motor errors. However, young adults may not reduce cocontraction as they adapt to standing or walking perturbations (6, 7).

Older adults often use more cocontraction across the whole body than young adults, presumably to increase limb stiffness to resist perturbations (8–10). During perturbed goal-directed reaching tasks, older adults did not reduce their motor errors or cocontraction as much as young adults (8, 11). Similarly, during postural and locomotor perturbations, older adults also used more cocontraction, indicating an increased effort to adapt to the perturbations (7, 9). An undesirable consequence of increased cocontraction during postural tasks is reduced balance performance, particularly in older adults (12). The increased cocontraction during balance tasks and walking in older adults seems to be an age-specific strategy, which is not due to a lack of sensory acuity and might be insufficient to respond to losses of balance (10, 12–15). Nonetheless, older adults can improve walking and balance performance and reduce cocontraction as they gain more experience with perturbations during postural tasks and walking (16, 17). However, these reductions in cocontraction may not translate to improved balance or walking metrics (18–20).

Reduction of muscle cocontraction and motor errors may not always be observed during adaptation to perturbations if alternative adaptation paradigms such as use-dependent learning are occurring. Use-dependent learning produces a prolonged adaptation of movements that does not wash out in a few trials or strides after removal of the perturbations (21). During use-dependent learning, perturbations do not directly hinder task completion. So, reducing motor task errors may not be necessarily advantageous to achieve the task goal. For example, applying brief belt accelerations at the toe-off of each leg on a split-belt treadmill would not challenge the balance of a walking person, such that subjects learn to increase the push-off force in response to perturbations and retain the stronger push-offs even after the perturbations are removed (22). The prolonged adaptation [which could correlate to increased behavior savings (23, 24)] can significantly boost rehabilitation performance. We recently showed that perturbing recumbent stepping with brief increases in resistance did not produce classic error-based adaptation but rather resulted in features of use-dependent learning in young adults (25). The brief resistive perturbations did not hinder the most explicit task goal of following a pacing cue. As such, subjects modified their stepping patterns without reducing temporal or spatial errors, and these modified patterns were sustained even after removal of the perturbations and stepping without perturbations for 2 min (25). Similarly, during perturbed cycling using a split crank that altered the relative phasing of the pedaling legs, subjects modified their muscle activity patterns and retained those patterns (26). The potential for shaping muscle activity, cocontraction, and motor behavior with use-dependent learning tasks has not been explored much.

The purpose of this study was to compare motor behavioral and muscular responses to perturbations during recumbent stepping, a task that elicits use-dependent learning, in young and older adults. To our knowledge, multimuscle coordination of a perturbed seated locomotor task has not been explored for older adults. Similarly, potential prolonged adaptation due to use-dependent learning has not been tested for older adults. We hypothesized that the motor behavioral responses of older adults would be similar to the young adult responses we observed previously (25). As such, we expected that the older adults would not show error-based adaptations. We also hypothesized that perturbations would increase cocontraction, which would be sustained after the perturbations were removed in young and older adults, consistent with use-dependent learning. Additionally, we hypothesized that older adults would exhibit more muscle cocontraction compared with young adults based on age-related increases in muscle cocontraction. We previously reported the motor behavioral activity of young adults (25), but the muscle activity or cocontraction data were not reported in that paper or previously published elsewhere. The older adult motor responses and muscle cocontraction data have not been published.

We used our robotic recumbent stepper to perturb young and older adults during recumbent stepping by briefly increasing the stepping resistance. Subjects completed four perturbed stepping tasks; each task involved a single perturbation that occurred at extension onset or midextension of the left or right leg. We instructed subjects to use both their arms and legs, but subjects could drive the stepper with only one limb as the recumbent stepper has only one degree of freedom. We recorded the stepping kinematics and the subject’s electromyograph (EMG) from 12 muscles and quantified motor errors, mean EMG, and the cocontraction index.

## METHODS

Seventeen young adults (11 females; age 25 ± 4.9 yr) and 11 older adults (4 females; age 68 ± 3.6 yr) participated in the study. Subjects were all right-handed based on which hand they would use to pick up an object from the floor. We were not able to recruit more older adult participants because of pandemic restrictions and the function loss of the hardware afterward. They self-reported no neurological impairments, no problems with their gait, no history of falls, and no broken bones for 2 yr before the data collection. Each participant also met the inclusion criteria based on four questionnaires to ensure that they could safely complete the experiment: *1*) Short performance battery (9/12) (27), *2*) Berg balance scale examination (50/56) (28), *3*) Mini Mental-State Examination (25/30) (29), and *4*) CHAMPS physical activity (30). The Institutional Review Board of the University of Central Florida approved the study, and subjects gave their written informed consent before starting the experiment.

### Hardware

**Figure 1.**
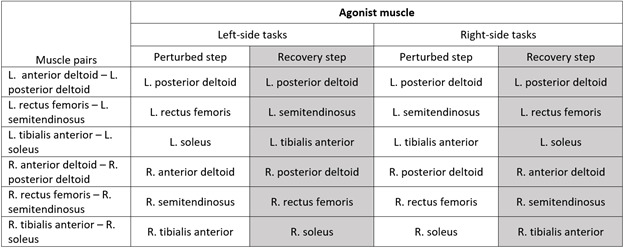
Schematic of the robotic recumbent stepper, perturbation types, and stepping blocks. *A*: the robotic recumbent stepper is a 1-degree of freedom stepping device with an integrated servomotor. The handles and pedals are mechanically coupled such that any limb can drive the stepping motion and move all the other limbs. A pacing cue of alternating black and white rectangles that were 180° out of phase with one another was projected on a screen in front of the subject. We did not include the signals from the biceps and triceps brachii muscles because of the required sensor change during the experiment. EMG, electromyograph. *B*: perturbations were brief increases in stepping resistance in the extension onset or midextension of each stride (shaded light green vertical rectangle). *C*: each task block consisted of 6 min of perturbed stepping padded by 2 min of unperturbed stepping at the beginning and end of the task. Random catch strides did not include a perturbation.

We used a recumbent stepper integrated with a servomotor (31) to introduce brief perturbations in the form of added resistance during stepping (Fig. 1*A*). The stepper (TRS 4000; NuStep, Inc., Ann Arbor, MI) was mechanically coupled so that the contralateral arm and leg would extend together. We used the servomotor’s position sensor (Kollmorgen, Radford, VA) to record the stepper’s kinematics at 100 Hz. Perturbations briefly increased stepping resistance for 200 ms. The magnitude of the resistance required 3× torque to drive the stepper at 60 steps per minute. Perturbations were applied once the targeted leg was at the extension onset or the midextension (Fig. 1*B*).

We used 12 wireless electromyography (EMG) sensors (Trigno; Delsys, Natick, MA) to record muscular activity at ∼1.1 kHz from the tibialis anterior, soleus, rectus femoris, semitendinosus, anterior deltoid, and posterior deltoid on both the left and right upper and lower limbs. After locating the sensor position according to the SENIAM guidelines (32), we abraded and cleaned the skin and attached the sensors with Delsys double-sided adhesive patches. Data streams of the EMG and stepper systems were synchronized with a trigger signal sent from the stepper controller to the EMG controller to start and stop recording simultaneously. We imported and preprocessed the stepper data in MATLAB (R2018b, MathWorks Inc, Natick, MA). We completed all EMG processing as well as stepping motor error quantification in Python 3.9, using NumPy 1.25 (33), SciPy 1.6 (34), Pandas 1.2 (35), Matplotlib 3.3 (36), and seaborn (37).

### Protocol

Data collection started with 2 min of quiet sitting followed by four 10-min stepping tasks and ended with another 2 min of quiet sitting. Each stepping task only included one perturbation *type*, i.e., 2 perturbation windows (extension onset or the midextension) × 2 legs = 4 perturbation types. The order of the perturbed trials was pseudorandomized. Each perturbed stepping task included three different *blocks* (Fig. 1*C*): *1*) pre: 2 min of unperturbed stepping at the start of each trial; *2*) perturbed stepping: 6 min of perturbed strides with a single perturbation type; *3*) post: 2 min of unperturbed stepping immediately after the end of the perturbed stepping period. The perturbed stepping block also included one random “catch” stride in every five perturbed strides, which did not apply a perturbation. We use “pre” and “preperturbation” interchangeably and also use “post” and “postperturbation” interchangeably.

We strapped the subject’s feet on the pedals, adjusted the seat position, and moved the handles to ensure that subjects would not lock their knees and could easily drive the stepper with the handles. Before each task, we instructed the subjects to *1*) step smoothly as if they were walking, *2*) use both their arms and legs to drive the stepper, and *3*) follow the pacing cues that were projected in front of them (Fig. 1). Pacing cues were set at 60 steps per minute to match older adults’ average walking pace (38) and were projected as two reciprocating black and white rectangles (Fig. 1). We did not provide any instruction on how to interpret the pacing cues. Subjects were given at least 2 min of training to become familiar with the pacing cues before the data collection started.

### Stepping Preprocessing and Stride Events

After importing the stepping data into MATLAB, we separated each task into blocks and strides. We defined a *stride* as the time from one extension onset of the perturbed leg to the next extension onset of the perturbed leg for each task. For each stride, we identified the following events: perturbed-leg extension onset, perturbation (start time), unperturbed-leg extension onset, and the end of the stride. We artificially inserted perturbation events to the unperturbed strides (i.e., pre, post, and catch strides) at the average latency of the perturbation events during the perturbed strides. We excluded any incomplete strides, which were the strides that did not include all the events.

### Motor Errors

**Figure 2.**
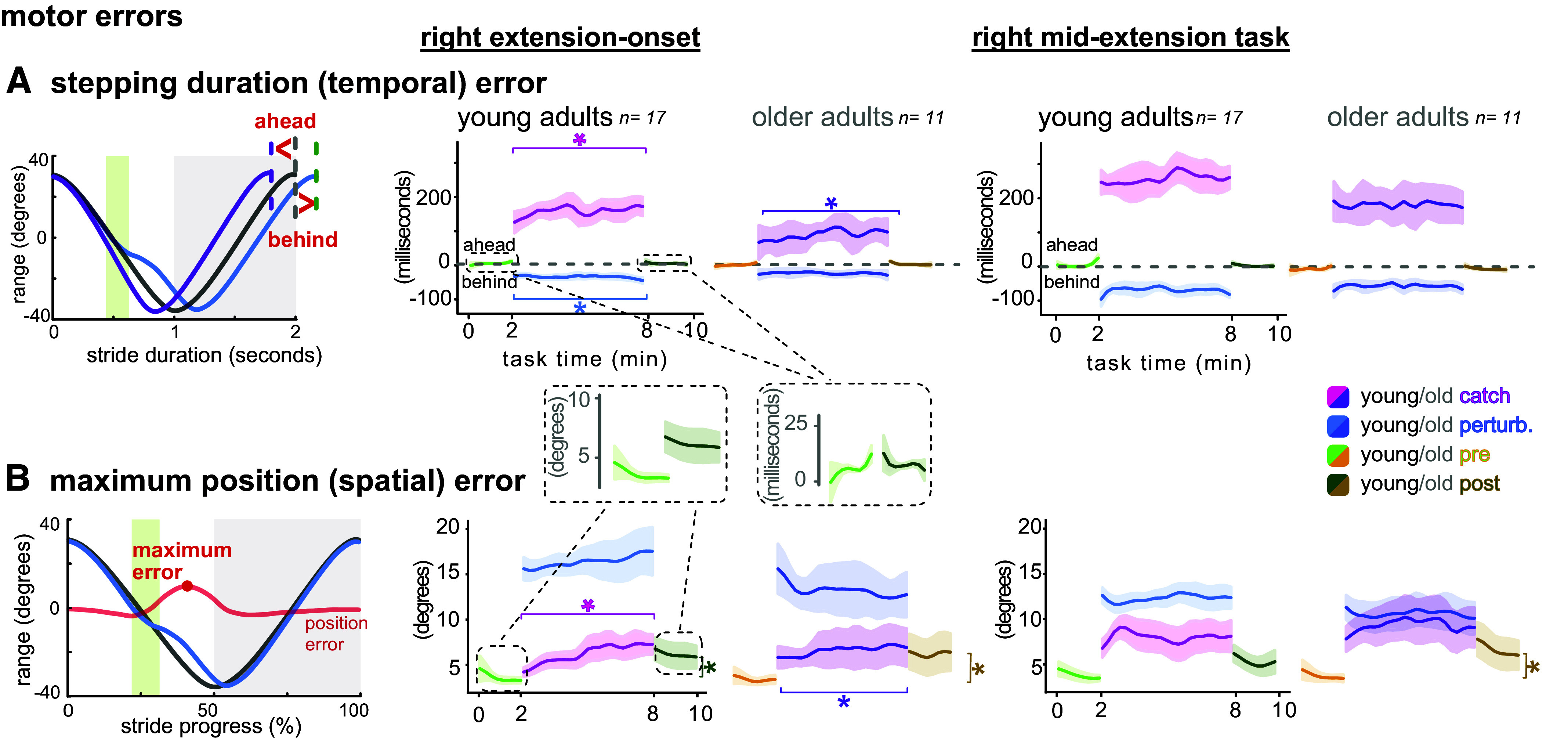
Schematic of motor errors and the motor error behavior for the right-side tasks. *Left*: the vertical light green rectangles indicate the perturbation periods. *Center* and *right*: the color-shaded areas in the behavior plots are the 95% confidence intervals. *P < 0.05. Horizontal brackets indicate significant differences from start to end. Vertical brackets indicate significant differences between end of pre and end of post. Overall, young and older adults presented similar behavioral responses, i.e., error levels and prolonged washout in response to the perturbations. *A*: the stepping duration (temporal) error was the difference between the duration of each step and the 2 s mark set by the pacing cue (gray line). Young and older adults could maintain their temporal errors <100 ms during the perturbed strides. *B*: the maximum position (spatial) error was the maximum difference between each stride’s profile and the average baseline (pre) stepping profile. Spatial errors for young adults for the perturbed and catch strides did not converge by the end of the perturbation period, whereas older adults trended to similar spatial errors by the end of the perturbation period. *Insets* show negligible pre-to-post temporal errors and significant change of the spatial errors from pre to post for young adults during the extension-onset task.

From the stepping kinematics, we quantified two motor error metrics, one temporal and one spatial. Based on the pacing cues at 60 steps per minute, subjects should have completed each stride in 2 s. We defined temporal error as the stride duration error, which was the difference between each stride duration and the 2 s (Fig. 2*A*). Because we instructed subjects to step smoothly, we expected the stepping profiles to be smooth and rhythmic during the preperturbation block. We defined spatial error as a stepping position error, i.e., the maximum difference of the time-normalized position profile during each stride from the averaged preperturbation stepping profile (Fig. 2*B*). Based on our hypothesis, we expected that both young and older adults would present similar temporal and spatial error trends across all tasks, including prolonged adaptation.

### EMG Processing

We imported and analyzed the EMG data in the Python environment using a custom processing pipeline based on Banks et al. (39). We resampled the EMG data to 1 kHz, band-pass filtered between 30 and 200 Hz, rectified, and low-pass filtered at 20 Hz to obtain the EMG linear envelopes. Filters were designed with the 6th-order Butterworth algorithm. We chose 20 Hz as the low-pass threshold to capture EMG fluctuations in response to our 200-ms perturbations (40). We then epoched and time-normalized the EMG data based on the stepping events for each stride. Finally, we normalized each muscle’s linear envelope to the overall average of the muscle’s linear envelope across all tasks.

We used the “fixed” approach to quantify cocontraction (39). We assumed that the agonist was the muscle that could drive the stepper without the activity of the other muscles. During the step that involved left leg extension, the left soleus, left rectus femoris, left posterior deltoid, right tibialis anterior, right semitendinosus, and right anterior deltoid act as functional agonists. The agonist muscles of the muscle pairs for each step are summarized in Table 1. The fixed cocontraction index (CCI) is calculated with the following equation:

$$\mathrm{CCI}=\frac{2\times I_{\mathrm{antagonist}}}{I_{\mathrm{agonist}}+I_{\mathrm{antagonist}}}$$

**Table 1.** Agonist muscles to drive the stepper for the left- and right-side tasks

Muscle Pairs	Agonist Muscle			
	Left-side tasks		Right-side tasks	
	Perturbed step	Recovery step	Perturbed step	Recovery step
Left anterior deltoid-left posterior deltoid	Left posterior deltoid	Left posterior deltoid	Left posterior deltoid	Left posterior deltoid
Left rectus femoris-left semitendinosus	Left rectus femoris	Left semitendinosus	Left semitendinosus	Left rectus femoris
Left tibialis anterior-left soleus	Left soleus	Left tibialis anterior	Left tibialis anterior	Left soleus
Right anterior deltoid-right posterior deltoid	Right anterior deltoid	Right posterior deltoid	Right posterior deltoid	Right anterior deltoid
Right rectus femoris-right semitendinosus	Right semitendinosus	Right rectus femoris	Right rectus femoris	Right semitendinosus
Right tibialis anterior-right soleus	Right tibialis anterior	Right soleus	Right soleus	Right tibialis anterior

**Figure 3.**
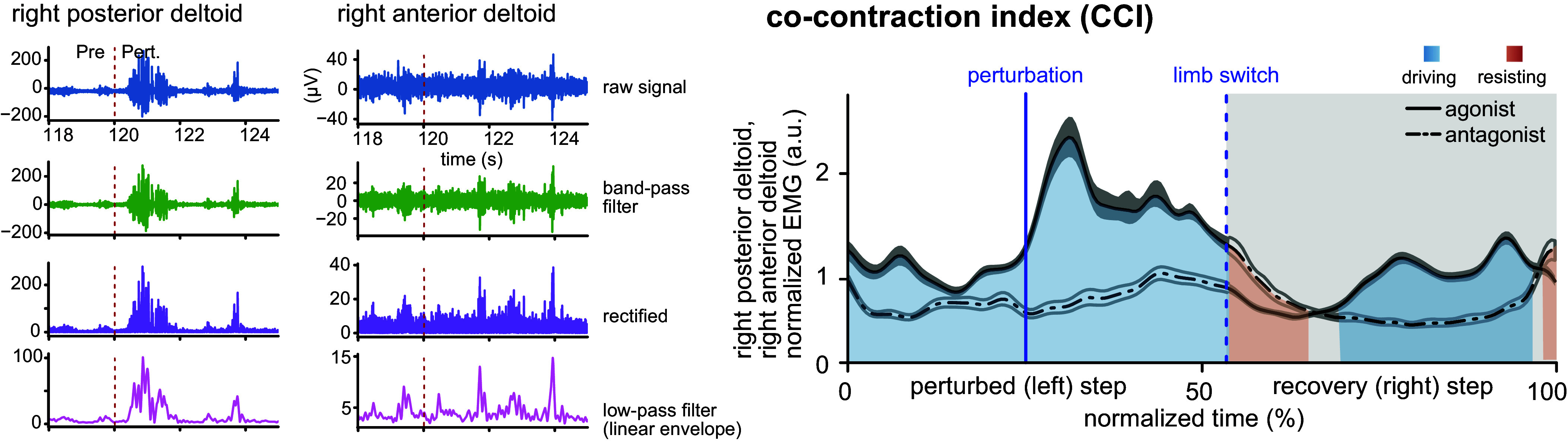
Exemplar electromyographic (EMG) signal of an agonist-antagonist muscle pair (the right anterior and posterior deltoid) during the right midextension perturbation task. *Left*: exemplary muscle electrical activity and the process of reaching the linear envelope. The red dashed line indicates the start of the perturbed stepping block of the task. *Right*: normalized normal envelopes of the 2 muscles with the sections of each step that correspond to driving (CCI < 1), and resisting (CCI > 1) modes. Based on the muscle pair role in the motion, CCI could be >, <, or = 1 for each step. a.u., Arbitrary units.

Here, *I*_antagonist_ and *I*_agonist_ are the integrals of the EMG linear envelopes over each step. Because of the stepper’s inherent redundancy, subjects may use a subset of muscle pairs, or even one, that could drive the stepper. In each step, this can be inferred from the CCI for that step (Fig. 3). CCI is usually expected to remain <1 (i.e., the blue area in Fig. 3 is greater than the red area), so the net activity of the muscle pair can drive the limb in the designated stepping direction. However, in our study, CCI might become >1 if the designated antagonist helps to control stepping while the agonist is not involved in driving the stepper. As such, CCI < 1 means that the muscle pair is mainly driving the stepping motion; CCI > 1 would mean that the muscle pair is resisting the motion; and CCI ≈ 1 means that the muscle pair either controls the motion (e.g., driving in some period and resisting in another period of a step) or is not active. To quantify the number of muscle pairs resisting the motion, we defined the resistance ratio as

$$\text{Resistance Ratio}=\frac{\text{No. of Resisting Muscle Pairs}}{\text{Total Muscle Pairs}}$$

### Statistical Analysis

Motor errors were quantified per stride, but CCI was quantified per step to allow for designation of agonist and antagonist muscles based on the direction of the motion. We used the SMART toolbox to report the errors and cocontraction values as continuous variables (41). The main advantages of using SMART over binning methods are that the varied number of strides would not affect the results and that each subject contributes equally to the overall average. Motor errors were first quantified 10 times per minute to present the error behavior in Fig. 2. Later, we quantified both CCI and motor errors per minute to quantify the intervals where the CCI was significantly greater or smaller than 1 and to compare motor errors and resistance ratio between young and older adults. The test on the CCI difference from 1 was performed with SMART’s one-sample bootstrapped *t* test, with the clustering technique to account for multiple comparisons.

Multiple comparisons and comparisons between young and older adults were performed with the Pingouin toolbox version 0.5.2 (42). For multiple comparisons, we used repeated-measures analysis of variance (rANOVA) followed by post hoc *t* tests with Tukey correction. We ensured that the rANOVA requirements (i.e., normal distribution, lack of outliers, and sphericity) (43) were met for the measurements using SPSS software (version 25.0; IBM Corp., Armonk, NY). These multiple comparisons were performed for motor errors at the start and end of each block. We used Student *t* tests after rejecting possible outliers for comparisons between young and older adults. We had a priori hypotheses for the muscular responses as the older adults would use more muscle pairs to resist the motion and have higher CCI than young adults. The alpha was set to 0.05 for all tests.

## RESULTS

### Temporal Error

Young and older adults did not reduce their temporal errors as they gained more experience with the perturbations, indicating a lack of error-based adaptation, but these did wash out after the perturbations were removed (Fig. 2*A*). Both young and older adults had ∼50-ms temporal errors during perturbed strides, whereas the temporal errors during catch strides were ∼150 ms (Fig. 2*A*). The rANOVA was significant for temporal errors in each task [young: *F*(6,96) > 144, *P* < 0.0005; older: *F*(6,60) > 15, *P* < 0.0005]. However, the post hoc tests only indicated significant and meaningful differences in the right extension-onset temporal errors at the start and end of catch strides for young adults (*P* = 0.003). Although young adults demonstrated a significant increase in temporal error from the start to the end of the perturbed strides during the right extension-onset task, the error was <50 ms, which would be imperceptible to the subject. Both young and older adults reduced their temporal errors to baseline levels after the perturbations were removed, indicating temporal error washout. The left side also showed a similar temporal error increase for left extension-onset catch strides for both young and older adults [young: *F*(6,96) > 144, *P* < 0.0005, post hoc *P* < 0.05; older: *F*(6,60) > 24, *P* < 0.0005, *P* < 0.05] (Supplemental Fig. S1a, see https://doi.org/10.6084/m9.figshare.25375738).

### Spatial Error

Spatial errors of older adults during catch and perturbed strides trended to similar levels by the end of the perturbed block, whereas there was not such a trend for young adults (Fig. 2*B*). Spatial errors for young adults during the catch strides were <10° for both perturbation tasks but were <20° for the right extension-onset perturbed strides and <15° for the right midextension perturbed strides. The difference between the spatial errors of catch strides and of perturbed strides for older adults was diminished toward the end of the right extension onset and not present during the right midextension perturbations. The rANOVAs were significant for the spatial errors of every task [young *F*(6,96) > 38, *P* < 0.0005; older *F*(6,60) > 17, *P* < 0.0005]. The post hoc tests showed that after removal of the perturbations spatial errors were always higher than pre levels for both young and older adults and did not wash out (post hoc, young and old *P* < 0.01). However, only young adults showed increased spatial errors during the right extension-onset catch strides (*P* < 0.0005). Similarly, rANOVAs were significant for the left-side tasks [young *F*(6,96) > 24, *P* < 0.0005; older *F*(6,60) > 12, *P* < 0.0005]. The spatial errors for young and older adults did not wash out and remained higher than the pre levels at the end of the left extension-onset or midextension task (post hoc, young *P* < 0.02, old *P* < 0.01; Supplemental Fig. S2b, see https://doi.org/10.6084/m9.figshare.25375747). Contrary to the right-side perturbations, only older adults showed increased spatial errors during the left extension-onset catch strides (*P* = 0.023).

### Muscle Cocontraction

**Figure 4.**
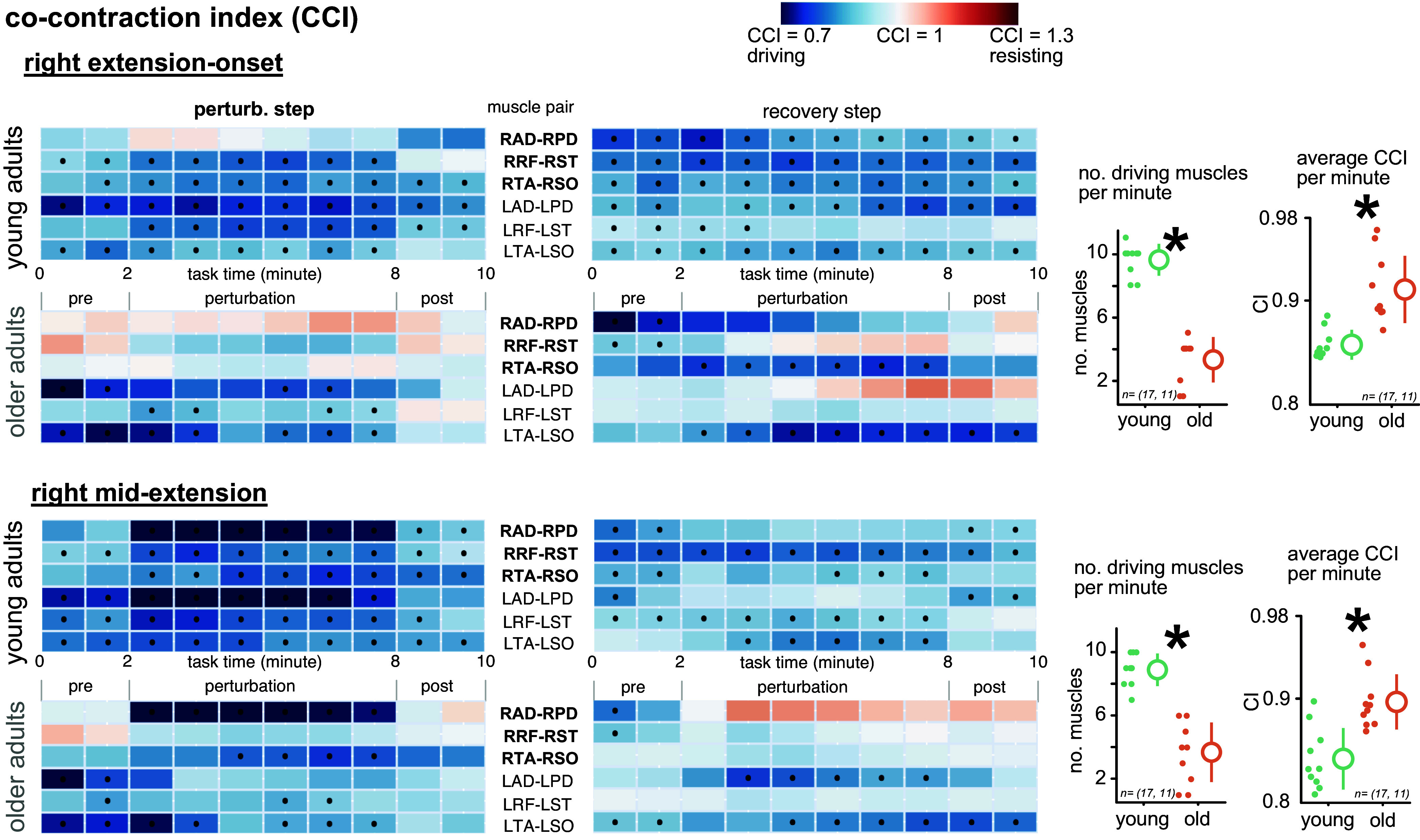
Cocontraction index (CCI) progress over task time for the right extension-onset and right midextension tasks. Heatmaps indicate the CCI per 1 min of stepping. Muscle pairs (L, left; R, right) are shown over the heatmap rows, and the CCIs for the perturbed step and the recovery step are separated and reported independently. Dots inside heatmap cells indicate a significant difference in the CCI from 1 (*P* < 0.05), suggesting that the muscle pair significantly contributed to driving (or resisting) the motion. Young adults used most of their muscle pairs to drive the stepper, whereas older adults only used a handful of their muscle pairs to drive the stepper. Older adults seemed to have fewer driving muscle pairs for the recovery step. Young adults used significantly more muscle pairs per minute to drive the stepper. Older adults exhibited greater CCI per minute during the tasks. *P < 0.05 with a priori. For graphs on *right*, small dots are individual values, larger dots are averages, and the bar is the SD. AD, anterior deltoid; PD, posterior deltoid; RF, rectus femoris; SO, soleus; ST, semitendinosus; TA, tibialis anterior; CI, confidence interval. *n* = (17, 11) indicates the comparison performed between 17 young adults and 11 older adults.

Young adults used most of their muscle pairs (∼10/12) to drive the stepper, whereas older adults only used a small subset of their muscle pairs (∼4/12) for driving the stepper (Fig. 4). Young adults tended to drive the stepper during the right extension-onset tasks with almost all their muscle pairs. This is shown in Fig. 4 with the blue-shaded heatmaps for the muscle pairs (indicating CCI > 1) over the course of the tasks and dots over the maps, confirming that CCI is indeed significantly greater than 1. Young adults did not use their right deltoid muscle pair and left thigh muscles [left rectus femoris (LRF)-left semitendinosus (LST)] for the right extension-onset task. Similarly, young adults started the right midextension task without using the right anterior deltoid (RAD)-right posterior deltoid (RPD) pair but incorporated this muscle pair as soon as the perturbations were introduced. Instead, during the recovery step of the right midextension task young adults did not tend to use their upper limb muscle pairs [both left anterior deltoid (LAD)-left posterior deltoid (LPD) and RAD-RPD] in the perturbation block. Older adults only used a small subset of the muscle pairs to drive the stepping device, as indicated by the failure of rejecting CCI = 1 (indicated by the absence of dots over the CCI heatmaps) for most of the muscles, as shown in Fig. 4. The shank muscle pairs [left (LTA-LSO) and right (RTA-RSO) tibialis anterior-soleus] seemed to drive the stepper most of the time before, during, and after the perturbations. Older adults also presented trends of resisting muscle pairs, especially during the recovery steps, but CCI was never significantly greater than 1. Both young and older adults did not use their right upper limb muscle pair (RAD-RPD) during the recovery. Overall, young adults used a significantly larger pool of muscle pairs to drive the stepping device per minute than older adults (*t* test *P* < 0.001). Also, older adults had significantly greater CCI per minute across their muscle pairs than young adults for all tasks (*t* test *P* < 0.001; Fig. 4). A similar trend can also be seen for the left-side perturbations, where young adults had a significantly larger pool of muscle pairs to drive the device than older adults (Supplemental Fig. S2). Looking at all four tasks, older adults seem to rely on their left shank muscle pair (LTA-LSO), with and without facing the perturbations, and also irrespective of movement direction.

### Young vs. Older Adult Motor Errors and Resistance Ratio

**Figure 5.**
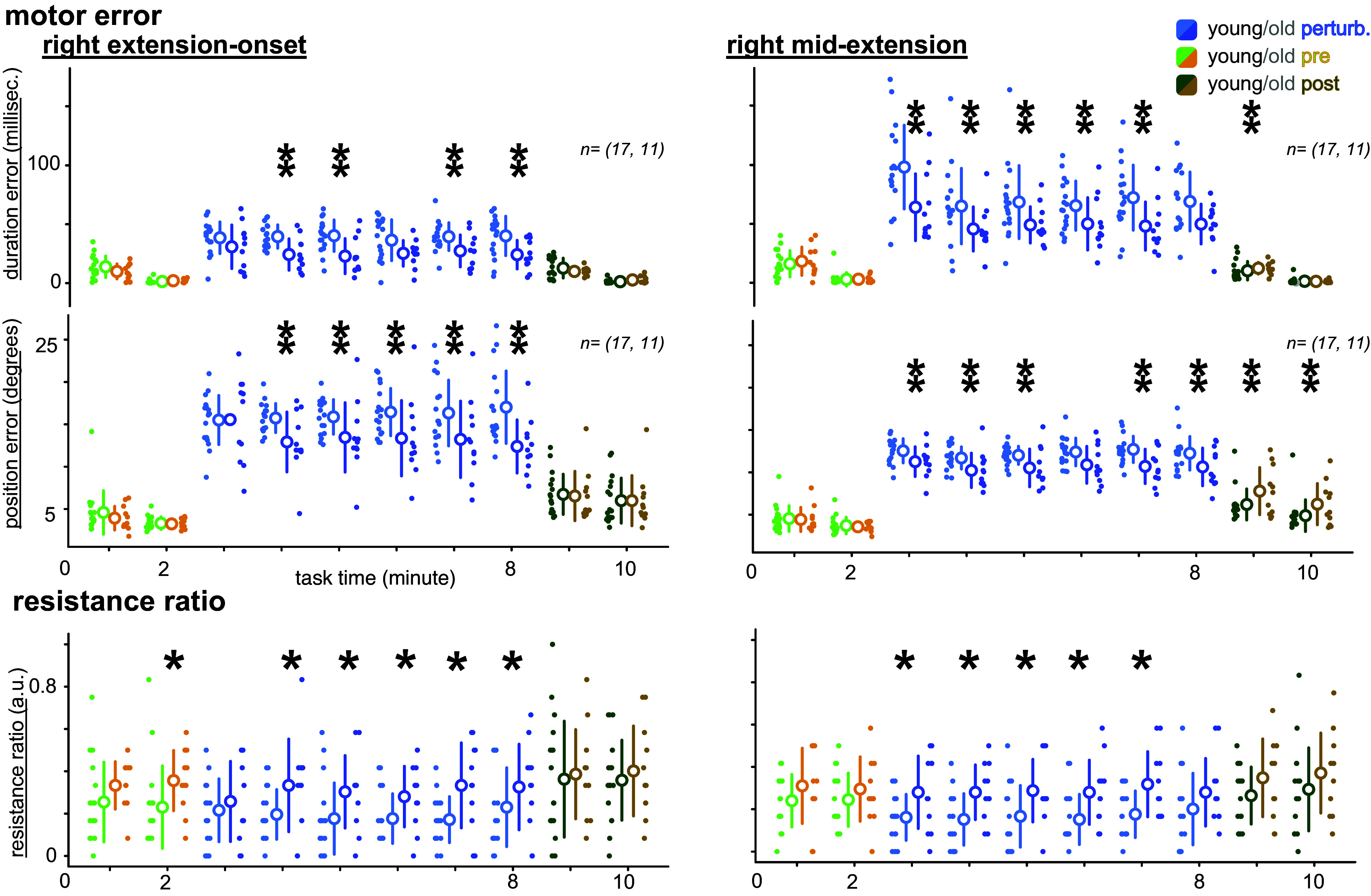
Comparison of motor errors and resistance ratio between young and older adults for the right extension-onset and right midextension tasks. Older adults demonstrated less temporal and spatial motor error during the perturbation block. However, older adults tended to have a greater resistance ratio (i.e., the ratio of the resisting muscle pairs to all muscle pairs) during the perturbation block. **P < 0.05 without a priori, *P < 0.05 with a priori. Small dots are individual values, larger dots are averages, and the bar is the SD. *n* = (17, 11) indicates the comparison performed between 17 young adults and 11 older adults.

Older adults had less temporal and spatial error and showed a greater resistance ratio, indicating that they had more muscle pairs controlling or resisting the motion over time (Fig. 5). Older adults consistently presented less duration (temporal) errors during right extension-onset and midextension perturbations than young adults. Similarly, older adults tended to have less position (spatial) error than younger adults for both perturbation types (Fig. 5, *t* test *P* < 0.05). Looking at the resistance ratio, older adults had overall more resisting muscle pairs during the perturbations than young adults, especially for the extension-onset tasks. The resistance ratio was never significantly different between young and older adults during the pre- or postperturbation blocks, but older adults demonstrated greater temporal and spatial errors during the right midextension postperturbation block (*t* tests *P* < 0.05). Similar trends were also present for the left-side perturbations, with even more significant resistance ratio differences between young and older adults during the perturbation block (Supplemental Fig. S3, see https://doi.org/10.6084/m9.figshare.25375744).

## DISCUSSION

We quantified and compared motor error behavior and muscle cocontraction of young and older adults responding to recumbent stepping perturbations. As expected, young and older adults retained prolonged motor modifications after the perturbations were removed, suggesting that use-dependent learning also occurred for older adults. Unlike young adults, spatial errors in catch and perturbed strides approached similar levels by the end of the perturbation block for older adults. Young adults used a larger pool of muscles than older adults to drive the stepper across all tasks. Older adults had overall greater CCI for all tasks, supporting our hypothesis of the influence of age on cocontraction levels. Also, the resisting cocontraction of older adults (reflected in the resistance ratio) generally increased during the perturbation block more than that of young adults. At the same time, older adults consistently had less motor error than young adults. Interestingly, after the perturbations, older adults tended to use only one muscle pair (LTA-LSO) to drive the stepper. Results suggest that although increased cocontraction can be expected with aging, older adults use their distinct muscle recruitment strategies to achieve similar or lower motor error levels than young adults.

The incorporation of use-dependent learning in response to the perturbation during recumbent stepping was shared between young and older adults. Motor errors did not decrease during the perturbed block for young or older adults, indicating that error-based adaptation did not occur. Instead of decreasing, the spatial errors were prolonged during the perturbed block and sustained through the post block in both young and older adults, which indicates use-dependent learning. The results suggest that, regardless of age, subjects perceived that following the pacing cue was their main goal in the perturbed stepping tasks and that modifying the stepping profile did not hinder achieving the task goal, which led to the retention of the modified stepping profile (21). The results suggest that use-dependent learning paradigms could be used across the age span as an effective way to alter motor behavior. We also found that cocontraction indexes (CCIs) did not likely decrease as subjects gained more experience with our perturbations (Fig. 4). In typical error-based adaptation studies, cocontraction often decreases with the adaptation (1, 44). Taken together, motor errors, CCI, and resistance ratio indicate that use-dependent learning occurred as subjects responded to perturbations applied on a stride-by-stride basis during recumbent stepping.

Older adults used fewer muscle pairs to drive the stepper and had a greater resistance ratio compared with young adults (Fig. 4 and Fig. 5). Recumbent stepping is a mechanically redundant task. As such, subjects can drive the stepper with just one muscle pair in one of the four limbs. Overall, older adults had 4 out of 12 muscle pairs driving the stepping motion compared with 10 out of 12 muscle pairs for young adults (Fig. 4), indicating that older adults used fewer resources to drive the stepper. Also, the resistance ratio trended greater for older adults during the perturbation block (Fig. 5). This aligns with previous studies of perturbed walking and perturbed balance indicating that older adults use fewer muscle synergies to respond to perturbations than young adults (45, 46). Overall, by increasing cocontraction to potentially increase limb stiffness, older adults seemed to be able to resist and reject the perturbations such that the older adults had less motor error compared with young adults during the perturbation block (Fig. 5).

Interestingly, older adults also used fewer muscle pairs to drive the stepper in the postperturbation block compared with the pre block. Young adults, however, presented the opposite behavior, in which they likely incorporated more muscle pairs during the post block than the pre block (Fig. 4). This contrast in muscle recruitment indicates that although both young and older adults successfully learned how to overcome the perturbations and retained their learned behavior after the perturbations were removed, they used two vastly different approaches to achieve this goal and tended to keep their learned muscle recruitment patterns during the postperturbation block. The results support the notion of use of cocontraction by older adults as a strategy to respond to motor perturbations (7, 8). Still, our results are in contrast with the previously reported results that such cocontraction would hinder older adults from adapting to the perturbations as much as young adults (8, 12). This decoupling of cocontraction and motor adaptation might be because of removal of the balance and fall risk from the recumbent stepping or due to the novel use-dependent learning paradigm that subjects implement with brief stepping perturbations. We have recently shown that perturbations during recumbent stepping engage several cortical areas, including the supplementary motor area and the anterior cingulate cortex (25). The age-dependent control strategies may suggest that older adults would not follow the same cortical dynamics as young adults in response to the perturbations. Furthermore, older adults’ active driving of stepping with the shank muscle pair (tibialis anterior-soleus) is a distinct muscle recruitment pattern compared with balance control, where older adults incorporate their hip muscles more than shank muscles in response to perturbations (47, 48). These findings are particularly important for age-specific and closed-loop rehabilitation, where reinforcing neural control to regain its “normal” state is the rehabilitation goal.

We made several assumptions in our analyses and quantification of cocontractions throughout this study. We did not use the commonly suggested EMG normalization method using the maximal voluntary contraction (MVC) (49, 50) because of significant prior research in the human locomotion domain suggesting that such normalization may increase the within- and between-subject variability (51–54). A drawback of not using MVC for normalization is losing the ability to compare effort between individuals, which was not required for this experiment. Furthermore, we used the fixed approach for computing cocontraction, assigning a specific role for each muscle in each step (Table 1). Other cocontraction quantification methods include assuming the less active muscle as the antagonist (55) and discounting agonist muscle activation by the antagonist muscle activity (i.e., wasted contraction) (1). Assuming the less active muscle as the antagonist does not align with the muscle roles in a complex movement, which could be as a facilitator (i.e., agonist) or as a hindrance (i.e., antagonist). Previous research (39) and our preliminary results also suggested that the wasted contraction method would not have provided additional benefits in this context. We used single-differential EMG electrodes for our data collection with an interelectrode distance of 10 mm. A previous study has found up to 17% cross talk for this electrode type during gait for lower limb muscles (56). We do not believe that the potential cross talk would affect the cocontraction analysis, as the agonist and antagonist muscles are usually located far from each other and there would be little chance of cross talk between them. Other limitations of this study include not incorporating force data and attributing the perturbations to the extending leg. The recumbent stepper is equipped with load cells for pedals and handles. However, we decided not to use the force and moment data for this study because the inertia of the device would contaminate the force data, especially during the perturbations. Although we asked subjects to use both arms and feet to drive the stepper, we attributed the perturbations to the extending leg. A previous study and our preliminary tests (not reported here) showed that the lower limb extension contributes the most to compensate for increased stepping resistance (57).

Perturbed recumbent stepping is a seated locomotor exercise that engages distinct control mechanisms in young and older adults. Whereas young adults used most of their muscle pairs to drive the stepper device and overcome the perturbations, older adults used only a handful of their muscle pairs to drive the stepper. Nevertheless, both groups were successful in having imperceptible temporal errors. The outcomes reinforce the notion of differentiable motor control mechanisms across age groups, which might stem from differences in the neural control of movement and should be considered in designing rehabilitation paradigms.

## DATA AVAILABILITY

Processed data and analysis code are available from github.com/neuromechanist/olderAdult_cocontraction.

## SUPPLEMENTAL MATERIAL

10.6084/m9.figshare.25375738 Supplemental Fig. S1: https://doi.org/10.6084/m9.figshare.25375738.

10.6084/m9.figshare.25375747 Supplemental Fig. S2: https://doi.org/10.6084/m9.figshare.25375747.

10.6084/m9.figshare.25375744 Supplemental Fig. S3: https://doi.org/10.6084/m9.figshare.25375744.

## GRANTS

This work was partially supported by the National Institute on Aging of the National Institutes of Health under R01AG054621.

## DISCLOSURES

No conflicts of interest, financial or otherwise, are declared by the authors.

## AUTHOR CONTRIBUTIONS

S.Y.S. and H.J.H. conceived and designed research; S.Y.S. performed experiments; S.Y.S. and H.J.H. analyzed data; S.Y.S. and H.J.H. interpreted results of experiments; S.Y.S. and H.J.H. prepared figures; S.Y.S. drafted manuscript; S.Y.S. and H.J.H. edited and revised manuscript; S.Y.S. and H.J.H. approved final version of manuscript.
